# Oilseeds from a Brazilian Semi-Arid Region: Edible Potential Regarding the Mineral Composition

**DOI:** 10.3390/foods9020229

**Published:** 2020-02-21

**Authors:** Ivone M. C. Almeida, M. Teresa Oliva-Teles, Rita C. Alves, Joana Santos, Roberta S. Pinho, Suzene I. Silva, Cristina Delerue-Matos, M. Beatriz P. P. Oliveira

**Affiliations:** 1 REQUIMTE/LAQV, Departamento de Ciências Químicas, Faculdade de Farmácia, Universidade do Porto, Rua Jorge Viterbo Ferreira, 228, 4050–313 Porto, Portugal; ivonemalmeida@gmail.com (I.M.C.A.); rcalves@ff.up.pt (R.C.A.); joanasantoscma@sapo.pt (J.S.); 2 REQUIMTE/LAQV, Instituto Superior de Engenharia do Porto, Instituto Politécnico do Porto, Rua Dr. António Bernardino de Almeida, 431, 4200–072 Porto, Portugal; mtt@isep.ipp.pt (M.T.O.-T.); cmm@isep.ipp.pt (C.D.-M.); 3 Laboratório de Recursos Econômicos e Fitoquímica, Departamento de Biologia, Área de Botânica, Universidade Federal Rural de Pernambuco, Av. Dom Manoel de Medeiros s/n, Dois Irmãos, CEP, Recife-PE 52171–900, Brazil; rsampaiop@gmail.com (R.S.P.); suzene@db.ufrpe.br (S.I.S.)

**Keywords:** oilseeds, Caatinga, native, minerals, spectrometry

## Abstract

Oilseeds from five native plant species with edible potential from the Brazilian Caatinga semi-arid region (*Diplopterys pubipetala*, *Barnebya harleyi*, *Croton adamantinus*, *Hippocratea volubilis*, and *Couroupita guianensis*) were investigated regarding their mineral contents. The minerals, Na, K, Ca, Mg, Fe, Cu, Cr, Al, were analyzed by high-resolution continuum source atomic absorption spectrometry (HR–CS AAS) and P by the vanadomolybdophosphoric acid colorimetric method. K, Mg, and P were the main elements found (1.62–3.7 mg/g, 362–586 µg/g, and 224–499 µg/g dry weight (dw), respectively). *B. harley* seeds contained the highest amounts of K and P, while *C. guianensis* seeds were the richest in Mg. Fe was the most abundant oligoelement (2.3–25.6 µg/g dw). Cr contents were below the limit of quantification for all samples and Al amounts were low: 0.04–1.80 µg/g dw. A linear discriminant analysis clearly differentiated *B. harleyi* and *C. guianensis* samples from the remaining ones. In sum, these oilseeds from the Brazilian Caatinga semi-arid region seem to have the potential to be used as natural sources of minerals, mainly K.

## 1. Introduction

The plant species that grow in the arid and semi-arid land regions of the world have attracted considerable attention in recent years. The Caatinga, the main ecosystem in the Northeastern Brazil (~800.000 km^2^), is a seasonally dry tropical forest composed by a heterogeneous mix of plant species, primarily deciduous xerophytic spiny shrubs, and trees [1,2]. Despite the huge diversity, only a few of the species of this semi-arid region are exploited for industrial purposes [2]. The rural populations living in this area use many of these plants for food, fuel, timber, medicines, and livestock feed. These products contribute to income generation and offer an alternative source of livelihood when crops fail under the erratic and low rainfall conditions. However, human intervention over the years, such as deforestation, livestock pasture, and cropping, has contributed to the loss of vegetation cover and biodiversity, and extensive soil degradation in this biome [3,4,5,6,7,8,9]. 

Although different studies have already identified some plant species with great economic potential [2,3,8,9,10,11], the lack of scientific knowledge for the majority remains. The evaluation of the chemical composition of non-conventional tropical plants is essential not only for their fundamental knowledge, but also to support their use as raw material for food, chemical, and pharmaceutical industries [5]. For instance, in a recent study, Andrade et al. showed that native species from Caatinga used by the local population to treat inflammatory disorders have also a good photoprotective potential and could be used for pharmaceutical preparations [11]. In addition, nuts and seeds from the tropics have been shown to be rich in lipids and relatively good sources of inexpensive and renewable carbohydrates and proteins, and compounds with high added value, like lipid-soluble vitamins, phytosterols, and other phytochemicals, as well as notable quantities of minerals [12,13,14]. 

The insufficiency of minerals in humans is mainly caused by an unbalanced diet. In fact, Fe, Ca, Mg, and Cu are among the mineral elements most commonly lacking in human diets [15], and their deficiency could have a negative impact on health. The information about the mineral composition of unconventional oilseeds from Caatinga is very scarce. The aim of this work was, therefore, to analyze the mineral profile of oilseeds from five native species of this region using high-resolution continuum source atomic absorption spectrometry (HR–CS AAS), in order to evaluate their edible potential as a source of minerals. The seeds selected for this study are from Brazilian plants traditionally used in folk medicine, namely, *Croton adamantinus* Mull. Arg. (Euphorbiaceae), *Barnebya harleyi* W.R. Anderson & B. Gates *Diplopterys pubipetala* (A. Juss.) W.R. Anderson & C. Davis, (Malpighiaceae), *Hippocratea volubilis* L. (Celastraceae), and *Couroupita guianensis* Aubl. (Lecythidaceae). The present work is, as far as we know, the first published report on the mineral composition of these oilseeds. 

## 2. Materials and Methods 

### 2.1. Chemicals and Standard Solutions

Ultrapure water from a Simplicity 185 system (resistivity 18.2 MΩ cm; Millipore, Belford, USA) and nitric acid (65%; Suprapure^®^, Merck, Darmstadt, Germany) were used for the preparation of samples and standards. All reagents were of analytical or suprapure reagent-grade. Working standard solutions were prepared from the stock solutions of Na, K, Ca, Mg, Fe, Cu, Cr, and Al (1000 mg/L; Panreac, Barcelona, Spain) by proper dilution with a 0.5% and 1% (*v*/*v*) nitric acid solution. Cesium chloride (0.1% *w*/*v*; Sigma-Aldrich, Steinheim, Germany) was used as flame ionization chemical suppressor. For Ca analyses, lanthanum chloride heptahydrate (1%; *w*/*v*) was added to all solutions in order to minimize the oxy–salts interferences in FAAS analysis. A 0.1% (*w*/*v*) amount of magnesium nitrate (10 g/L; Panreac, Barcelona, Spain) was used as matrix modifier for the electrothermal atomic absorption spectrometry (ETAAS) quantification of Fe and Al; 0.05% (*w*/*v*) magnesium and 0.1% (*w*/*v*) palladium nitrates (10 g/L; Merck, Darmstadt, Germany) for Cu analysis. 

Potassium dihydrogen phosphate (99.5%; Riedel-de Haën, Seelze, Germany), ammonium heptamolybdate tetrahydrated (99.0%, Merck, Darmstadt, Germany), and ammonium monovanadate (99.0%, Merck, Darmstadt, Germany) were the reagents used for P analysis. Working standards solutions were manually prepared for HR–CS FAAS and P analysis. Solutions for HR–CS ETAAS calibration curves were automatically prepared by the autosampler. 

All glassware and plastic materials were cleaned by treatment with nitric acid (10%, *v*/*v*) for 24 h and then rinsed with water, prior to use.

### 2.2. Samples

Mature fruits of five different indigenous plant species (*Diplopterys pubipetala*, *Barnebya harleyi*, *Croton adamantinus*, *Hippocratea volubilis*, and *Couroupita guianensis*) were obtained from five individuals of each species in Pernambuco State (Brazil). 

The fruits were dehydrated at 60 °C for 48 h, until constant weight, and seeds were manually removed from the fruits. Seeds of each species were thinly ground in a mill and stored in plastic containers at 4 °C until use. 

### 2.3. Sample Preparation

Aliquots (1 g) of the dried ground seed specimens were dry-ashed in a muffle furnace at 500 °C, overnight. To the resulting white ash, 200 µL of nitric acid (65%) was added and ashed for another hour at 500 °C. An amount of 5.0 mL of nitric acid (65%) was used to re-dissolve minerals and, afterward, evaporated to a final volume of 2 mL. After filtration, the solutions were diluted to 50 mL with ultrapure water. Blanks were also carried out. The mineralization procedure was done in triplicate for each composite sample [16].

### 2.4. Analytical Methodologies 

An Analytik Jena contrAA 700 atomic absorption spectrometer (Analytik Jena, Germany), equipped with a high-intensity xenon short-arc lamp as continuum radiation source, and automatic MPE60 and AS52s autosamplers (Analytik, Jena, Germany), for electrothermal and flame atomization, respectively, was used throughout this work. Air-acetylene flame (high-purity grade) was used for K, Mg, Ca, and Na quantification at 766.4908 nm, 285.2125 nm, 422.6728 nm, and 588.9953 nm, respectively. Transversely heated graphite furnace with platform-pyrolytically coated graphite tubes (Analytik Jena, Germany) and high-purity argon was used for electrothermal determinations of Fe (248.3270 nm), Cu (324.7540 nm), Cr (357.8687 nm), and Al (309.2713 nm). Pyrolysis and atomization temperatures were optimized to maximize absorbance signals and to minimize backgrounds and matrix interferences. The optimized electrothermal programs used are shown in Table 1. Measurements were performed at the selected primary atomic lines at a wavelength-integrated absorbance equivalent to 3 pixels (central pixel ± 1).

The method accuracy was assessed through recovery experiments of the seed samples spiked with three different concentrations (50%, 100%, and 125% of the sample contents) of the aqueous standards. Precision was evaluated through intra- and inter-day measurements, by performing three sample measurements on the same day (intra-day) and over three days (inter-day). The limits of quantification (LOQ) were based on the residual standard deviation (10-fold) of calibration curves. 

*P* was determined by the vanadomolybdophosphoric acid colorimetric method according to the Standard Method 4500-P [16]. The color development reagent for P determination was prepared by the addition of ammonium heptamolybdate tetrahydrated and ammonium monovanadate. Phosphorus measurements were performed at 420 nm in a dual-beam UV/Vis spectrophotometer (Evolution 300, Thermo Scientific, Boston, MA, USA). 

### 2.5. Statistical Analysis 

The results were reported as mean ± standard deviation. One-way analysis of variance (ANOVA) and Tukey’s HSD post-hoc test were applied to evaluate the possible significant differences (*p* < 0.05) in mineral content between the different seeds. The mineral compositions of the seeds were also compared using multivariate statistical analysis. A linear discriminant analysis (LDA) was performed with forward stepwise analysis, considering a *p*-value of 0.01, using Wilk’s lambda (λ) as the selection criterion, and an F-statistic value to determine the significance of the changes λ when a new variable is tested. Followed this was canonical analysis to retrieve more information about the nature of the discrimination between the samples. Statistical analysis was carried out using STATISTICA software v.10 for Windows (Statsoft Inc., Tulsa, USA).

## 3. Results and Discussion

In previous research, Pinho et al. already identified *D. pubipetala*, *B. harleyi*, *C. adamantinus*, and *H. volubilis* as promising species for cultivation, based on their oil content and fatty acids profile [13]. *C. adamantinus* is used in the treatment of wounds, due to its anti-inflammatory and analgesic properties. Their seeds contain 37.1% of oil, with high concentrations of essential linoleic (44.2%) and linolenic (45.2%) fatty acids, and are rich in vitamin E, interesting characteristics for the human diet [13,17]. *B. harleyi* and *D. pubipetala* seeds contain high amounts of protein and are promising sources of oil (46.4% and 45.3%, respectively), in particular, of α-linolenic acid (42.8% and 31.9%, respectively) [13]. *H. volubilis* is a traditional expectorant. The seeds contain edible oil (45%–50%), but with a slightly bitter taste. *C. guianensis* leaves, flowers, and barks are known to contain many active ingredients that possess antibiotic, antifungal, antiseptic, antinociceptive, and immunostimulant activities ([18,19,20]. They contain about ~30% of oil, consisting mainly in linoleic acid (>80%) [13,21,22]. 

The minerals analyzed in this work were selected based on their physiological importance (e.g., Ca, Mg, K, …) or relevance as eventual indicators of environmental contamination (e.g., Cr and Al), since the perception of their composition is essential to better understand their potential to be used in human and/or animal nutrition or, for instance, in the dietary supplement industry.

Flame (FAAS) and electrothermal atomic absorption spectrometry (ETAAS), inductively coupled plasma optical emission spectrometry (ICP–OES), and inductively coupled plasma mass spectrometry (ICP–MS) are the main methods employed in mineral analysis [23]. In this work, the more recent technique HR–CS AAS was employed to evaluate the mineral content of the referred seeds. This equipment presents main advantages over the traditional line source AAS, namely the visualization of the spectral environment in the vicinity of the analytical line, the simultaneous background correction, and the improvement in precision and detection limits due to the higher signal-to-noise ratio [24,25].

### 3.1. Method Validation

Method parameters including linear range and correlation coefficient (*r*), LOQ values, repeatability, and mean recovery percentages are presented in Table 2. Na, K, Ca, Mg, P, Fe, Cu, Cr, and Al were quantified in seeds by the external calibration method with aqueous standard solutions. Good linearity was achieved for all elements over the range of tested concentrations, with correlation coefficients higher than 0.997 for all calibration plots. The LOQ ranged from 4.6 (Ca) and 11 µg/g (Ca) for major elements, and from 0.025 (Cu) to 0.11 µg/g (Al) for minor elements. Accuracy and precision of the method were good: recoveries ranged from 85% to 114%, intra-day precisions from 1.5% to 5.2%, and inter-day relative standard deviation (RSD) between 2.1% and 7.9%. These data confirmed the suitability of the method for the designated application.

### 3.2. Seed Analysis

The potential of the selected oilseeds as sources of minerals (Na, K, Ca, Mg, P, Fe, Cu, Cr) was ascertained and is described in Table 3. 

Physiologically, the macroelements Na, K, Ca, and Mg are essential for several human body functions, while P is the principal reservoir for metabolic energy and a co-factor of many enzymes [26]. K was the most prevalent element in the mineral composition of these seeds, followed, in decreasing order, by Mg, P, Ca, and Na. Among the studied samples, *B. harleyi* contained the highest K concentration (3649 µg/g). K contents ranged from 1636 to 3649 µg/g, levels considerably higher than those reported by Onyeike and Acheru [27] for castor seeds, coconut seeds, dikanut seeds, groundnut seeds, melon seeds, oil bean seeds, and palm kernel seeds (134–168 µg/g). This is an interesting characteristic taking into account the health importance of K. 

The Mg contents in the samples (362 to 586 µg/g) were very modest compared to the levels found for some common culinary nuts and seeds (almonds (~2000 µg/g), pecans (~1500 µg/g), hazelnuts (2000 µg/g) [28], and mustard oilseeds (~7000 µg/g) [29]. 

The P content ranged from 224 to 499 µg/g. These results are lower than those reported for umbu (*Spondias tuberosa* Arr. Cam) seeds (7720–8250 µg/g) [10] and baru (*Dipteryx alata* Vog.) almonds (7300 µg/g) [30], two Brazilian plant species. 

Ca levels (134 to 227 µg/g) were lower than those reported for pumpkin seeds (390–420 µg/g), sunflower seeds, and pistachios (~900 µg/g), and peanuts (520–530 µg/g) [28], but seeds from *D. pubipetala*, *B. harleyi*, and *H. volubilis* presented Ca values identical to pine nuts (130 µg/g) [28]. The richest source of Ca were the seeds of *C. guianensis* (227 µg/g), with levels slightly higher to those found in the nuts of *Cyperus esculentus* (188 µg/g) [31]. 

The Na content was low for all the samples (<106 µg/g), another good characteristic for edible seeds having in view its health effects.

Fe and Cu are essential trace elements. About two-thirds of the total body iron are involved in metabolic or enzymatic functions, mostly in erythrocytes (hemoglobin) and muscles (myoglobin) while the physiological functions of Cu arise directly from its role in a number of Cu containing enzymes, being essential for brain growth and maturation. Cr is also an essential element usually present in food in the trivalent form. In living organisms, Cr (III) is a component of enzymes that controls glucose metabolism and synthesis of fatty acids and cholesterol. Cr (VI) is toxic and carcinogenic to humans, but it is not usually present in food [32,33]. 

From a food safety perspective, Al contents of the oilseeds were also evaluated. Most of the population is not at risk for Al toxicity since humans have diverse mechanisms to prevent significant absorption and to aid its elimination. However, when protective gastrointestinal mechanisms are bypassed, renal function is impaired or exposure is high, Al can accumulate in the body, and originate impaired neurological development, Alzheimer’s disease, metabolic bone disease, dyslipidemia or even genotoxic activity [34].

The levels obtained for the minor minerals Cu, Cr, and Al were generally low for all samples. Fe was the most abundant of the analyzed oligoelements in all seeds, ranging from 2.34 µg/g in *C. adamantinus* to ten-fold more (25.6 µg/g) in *B. harleyi*. These values are lower than those described for mustard seeds (75–170 µg/g) [29], but the Fe level in *B. harleyi* is comparable to the values found in the literature for some well-known edible nuts such as pecan nuts (24–26 µg/g) and peanuts (20 µg/g) [28], and to the tropical babassu (*Orbignya speciosa*) nuts (18–33 µg/g) and sapucaia (*Lecythis pisonis*) nuts (21–36 µg/g) [14]. *H. volubilis* and *C. guianensis* oilseeds contained similar Fe concentrations to those reported for cupuassu (*Theobroma grandiflorum*) seeds (7.5 µg/g) [14]. *D. pubipetala* and *H. volubilis* seeds contained less than quantifiable levels of Cu (<0.025 µg/g) and the remaining samples contained lower amounts than those found in mustard seeds (6–13 µg/g). Cr concentrations fell below its quantification limit (<0.039 µg/g) in all seeds. Aluminum concentrations ranged from 0.044 µg/g in *D. pubipetala* to 1.80 µg/g in *C. guianensis* seeds, less than the concentration indicated for *Allantoma lineata* seeds (8 µg/g), an Amazon tree from the same family [22]. 

In sum, *B. harley* seeds had the highest content of K, P, Na, Fe, and Cu, while *C. guianensis* presented the highest levels of Mg, Ca, and Al from the five analyzed seeds.

Although all the oilseeds analyzed showed, in general, a similar mineral profile (high concentration of K followed by Mg > P > Ca > Na > Fe > Al > Cu), their composition showed several significant (*p* < 0.05) differences between them (Table 3). A Linear Discriminant Analysis (LDA) was performed to determine which minerals were able to discriminate between two or more naturally occurring groups (Table 4). 

**Figure 1:**
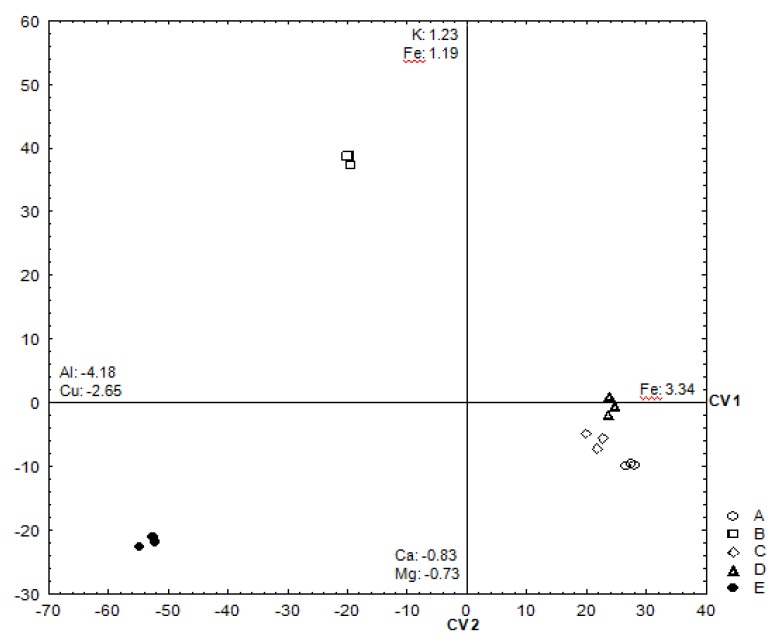
Scatterplot of all analyzed cases on the first canonical variate (CV1) versus the second canonical variate (CV2) (A: *Diplopterys pubipetala*; B: *Barnebya harleyi*; C: *Croton adamantinus*; D: *Hippocratea volubilis*; E: *Couroupita guianensis*).

The model was generated through forward stepwise analysis, considering variables one by one, choosing those with the most significant contribution to the data discrimination. Cr content was not considered in this multivariate analysis, as it was always inferior to the LOQ of the analytical method. All the other minerals (K, Mg, P, Ca, Na, Fe, Cu, and Al) were selected for the model generated by the LDA, Al and Fe being the variables that showed the highest contribution to the model. A subsequent canonical analysis generated, in total, four canonical variates (CV). The minerals and the case samples were displayed in a canonical variate scatterplot (Figure 1) of the first two discriminant functions, which comprised 98.9% of the information contained in the generated model. The first dimension (CV 1) represents 70.0% of the data variance, being more related to their Al, Fe, and Cu content. The second canonical variate (CV 2) adds more than 28.9% of information to the model, separating the samples according to their Fe, K, Ca, and Mg content. The relations of the variables with the first two canonical variates are presented by the resulting equations:

CV 1 = −4.18[Al] + 3.34[Fe] − 0.97[Mg] − 0.048[P] + 1.29[Ca] − 2.65[Cu] − 1.73[K] − 0.81[Na](1)

CV 2 = −0.15[Al] + 1.19[Fe] − 0.73[Mg] + 0.13[P] + 0.83[Ca] + 1.06[Cu] + 1.23[K] + 0.43[Na](2)

From the graphical display of the studied cases (see Figure 1), there were two seed species, namely *B. harleyi* and *C. guianensis*, visibly separated from the others. In the CV1, the Al content of the samples allowed to clearly distinguish the separation between the *C. guianensis* seeds and the others. The *B. harleyi* seeds were discriminated by their highest content of Fe, K, and Al content. The seeds *D. pubipetala*, *C. adamantinus*, and *H. volubilis* were displayed more closely to the positive side of the CV1 showing that the mineral composition of these three seeds was more similar.

## 4. Conclusions

The mineral contents of oilseeds from five native plant species from Brazil were evaluated. All the samples analyzed seem to be good sources of K, Mg, and P and presenting also low levels of Na. Nevertheless, *B. harley* oilseeds presented significantly higher amounts (*p* < 0.05) of K and P, Fe, and Cu compared with the other species. *C. guianensis* seeds were the best source of Mg. These two samples were, indeed, clearly differentiated from the remaining ones through linear discriminant analysis. In general, Cr and Al contents were low for all samples. The results show that these oilseeds from the Brazilian Caatinga semi-arid region have the potential to be used as food products and as mineral sources (mainly K).

## Figures and Tables

**Table 1:** Temperature programs for Fe, Cu, Cr, and Al determination in seeds by high-resolution continuum source electrothermal atomic absorption spectrometry (HRCS ETAAS).

Temperature (Ramp Time, Hold Time); °C (°C/s, s)				
-	Fe	Cu	Cr	Al
**Drying**	90 (3, 23.3)	90 (3, 23.3)	90 (3, 23.3)	90 (3, 23.3)
**Pyrolysis**	950 (300, 12.0)	900 (300, 11.8)	1300 (300, 13.2)	1400 (300, 13.5)
**Atomization**	1900 (1500, 4.6)	1800 (1500, 4.6)	2400 (1500, 4.7)	2450 (1500, 3.7)
**Cleaning**	2450	2450	2450	2500

**Table 2:** Methods validation parameters: linear range and correlation coefficients (*r*) of the analytical calibration curves, intra- and inter-day precisions (RSD, %), and limits of quantification (LOQ) for the elements studied.

Element	Wavelength (nm)	Detection	Calibration	*r*	LOQ	Precision RSD ^a^ (%)		Recovery ^b^
-		-	-	-	(µg/g)	Intra-Day	Inter-Day	(%)
Na	588.9953	HR-CS FAAS	0.1–1 (mg/L)	0.9992	8.4	1.6	2.4	85
K	766.4908	HR-CS FAAS	0.1–1 (mg/L)	0.9992	8.6	1.5	2.3	112
Ca	422.6728	HR-CS FAAS	0.1–1 (mg/L)	0.9996	4.6	3.5	4.3	112
Mg	285.2125	HR-CS FAAS	0.1–1 (mg/L)	0.9997	6.5	3.4	4.2	103
P	420.0	UV/Vis	0.1–3 (mg/L)	0.9995	11	1.6	2.1	104
Fe	248.3270	HR-CS ETAAS	3–15 (µg/L)	0.9976	0.090	5.1	6.3	93
Cu	324.7540	HR-CS ETAAS	3–15 (µg/L)	0.9999	0.025	3.7	4.3	106
Cr	357.8687	HR-CS ETAAS	3–15 (µg/L)	0.9998	0.039	5.2	7.9	110
Al	309.2713	HR-CS ETAAS	0.1–40 (µg/L)	0.9999	0.11	3.4	4.8	114

^a^ Average RSD: inter-day (*n* = 3); intra-day (*n* = 3, three days).; ^b^ Mean recovery of experiments with three levels of sample spiking (50%, 100%, and 125% of the sample contents).

**Table 3:** Mineral concentrations (µg/g dry weight (dw)) of the analyzed oilseeds.

Element	Species				
	*Diplopterys pubipetala*	*Barnebya harleyi*	*Croton adamantinus*	*Hippocratea volubilis*	*Couroupita guianensis*
Na	38 ± 1 ^c^	106 ± 6 ^a^	90 ± 12 ^ab^	74 ± 14 ^b^	8 ± 1 ^d^
K	1636 ± 135 ^c^	3649 ± 215 ^a^	1719 ± 282 ^c^	2554 ± 89 ^b^	1732 ± 187 ^c^
Ca	134 ± 6 ^c^	134 ± 9 ^c^	147 ± 14 ^cb^	170 ± 2 ^b^	227 ± 12 ^a^
Mg	447 ± 13 ^b^	494 ± 39 ^b^	362 ± 0.4 ^c^	384 ± 30 ^c^	586 ± 12 ^a^
P	288 ± 55 ^c^	499 ± 30 ^a^	397 ± 1 ^b^	224 ± 36 ^c^	419 ± 4 ^ab^
Fe	3.69 ± 0.03 ^c^	25.6 ± 2.5 ^a^	2.34 ± 0.23 ^c^	6.88 ± 0.19 ^b^	7.50 ± 0.54 ^b^
Cu	<LOQ	0.77 ± 0.15 ^a^	0.046 ± 0.002 ^b^	<LOQ	0.043 ± 0.005 ^b^
Cr	<LOQ	<LOQ	<LOQ	<LOQ	<LOQ
Al	<LOQ	1.00 ± 0.14 ^b^	<LOQ	0.192 ± 0.017 ^c^	1.80 ± 0.08 ^a^

Values are presented as mean ± standard deviation of triplicate determinations. Within each line, different letters represent significant differences between species, at *p* < 0.05.

**Table 4:** Summary of discriminant function analysis.

-	Wilks’ Lambda	Partial Lambda	F-Remove (4,4)	*p*-Level	Toler.
Na	0.000	0.163	3.840	0.149	0.173
K	0.000	0.302	1.737	0.339	0.333
Ca	0.000	0.205	2.910	0.203	0.290
Mg	0.000	0.557	0.597	0.692	0.289
P	0.000	0.391	1.168	0.468	0.732
Fe	0.000	0.094	7.270	0.067	0.069
Cu	0.000	0.125	5.265	0.102	0.106
Al	0.000	0.016	45.689	0.005	0.056

Wilks’ Lambda: 0.00000 approx. F (28.15) = 56.326, *p* < 0.0000.
